# Association of the *SPTLC3* rs364585 polymorphism and serum lipid profiles in two Chinese ethnic groups

**DOI:** 10.1186/s12944-016-0392-3

**Published:** 2017-01-05

**Authors:** Qing-Hui Zhang, Rui-Xing Yin, Hui Gao, Feng Huang, Jin-Zhen Wu, Shang-Ling Pan, Wei-Xiong Lin, De-Zhai Yang

**Affiliations:** 1 Department of Cardiology, Institute of Cardiovascular Diseases, The First Affiliated Hospital, Guangxi Medical University, 22 Shuangyong Road, Nanning, Guangxi 530021 People’s Republic of China; 2 Department of Pathophysiology, School of Premedical Sciences, Guangxi Medical University, Nanning, Guangxi 530021 People’s Republic of China; 3 Department of Molecular Biology, Medical Scientific Research Center, Guangxi Medical University, Nanning, Guangxi 530021 People’s Republic of China

**Keywords:** Lipids, Serine palmitoyl-transferase long-chain base subunit 3, Single nucleotide polymorphism, Environmental factors

## Abstract

**Background:**

Little is known about the association of the single nucleotide polymorphism (SNP) of rs364585 near serine palmitoyl-transferase long-chain base subunit 3 gene (*SPTLC3*) and serum lipid profiles. The present study was detected the association of the *SPTLC3* rs364585 SNP and several environmental factors with serum lipid profiles in the Han and Jing populations.

**Methods:**

Genotyping of the *SPTLC3* rs364585 SNP was performed in 824 unrelated individuals of Han and 783 participants of Jing by polymerase chain reaction and restriction fragment length polymorphism combined with gel electrophoresis, and then confirmed by direct sequencing.

**Results:**

There was no significant difference in either genotypic or allelic frequencies between Han and Jing, or between males and females of the both ethnic groups. The levels of serum low-density lipoprotein cholesterol (LDL-C) and the ratio of apolipoprotein (Apo) A1 to ApoB in Han; and total cholesterol (TC), high-density lipoprotein cholesterol (HDL-C) and LDL-C in Jing were different between the A allele carriers and the A allele non-carriers (*P* < 0.05-0.001). Subgroup analysis according to sex showed that the levels of LDL-C in Han males; TC and LDL-C in Jing males; and HDL-C and LDL-C in Jing females were different between the A allele carriers and the A allele non-carriers (*P* < 0.05-0.001), the A allele carriers had higher LDL-C and TC levels, and lower HDL-C levels than the A allele non-carriers. Serum lipid traits were also associated with several environmental factors in the Han and Jing populations, or in males and females of the both ethnic groups.

**Conclusions:**

The present study demonstrates the association between the *SPTLC3* rs364585 SNP and serum TC, HDL-C and LDL-C levels in our study populations. These associations might have ethnic- and/or sex-specificity.

**Trial registration:**

Retrospectively registered.

## Background

Cardiovascular disease (CVD) is the leading cause of morbidity and mortality worldwide [1, 2]. The increased incidence of CVD in the world has been linked to dyslipidemia [3–6]. Unfavorable lipid profiles including high levels of serum total cholesterol (TC) [7], triglyceride (TG) [8], low-density lipoprotein cholesterol (LDL-C) [9] and apolipoprotein (Apo) B [10], and low levels of high-density lipoprotein cholesterol (HDL-C) [11] and ApoA1 [10] play a significant role for CVD, and are the main target for therapeutic intervention. Epidemiological studies have consistently showed that dyslipidemia is a complex trait resulted from the joint effects of multiple genetic and environmental factors [12–14]. The heritability estimates of the interindividual variations in serum lipid levels from both twin and family studies are in the range of 40–70%, suggesting a considerable genetic contribution [15, 16]. Therefore, the understanding of the association of single nucleotide polymorphisms (SNPs) and serum lipid levels has become crucial in the pursuit of reducing CVD [17].

Recently, several genome wide association studies (GWAS) have reported the association of many SNPs near the serine palmitoyl-transferase long chain base subunit 3 gene (*SPTLC3*; also known as: *LCB3*, *SPT3*; Gene ID: 55304; OMIM:611120; chromosomal location: 20p12.1) with one or more lipid traits [18–20] though the biological function of *SPTLC3* in lipid metabolism is unknown. So far, the well-known function of *SPTLC3* is that it encodes a functional subunit of the SPT enzyme-complex that catalyzes the first and rate-limiting step of de novo sphingolipid synthesis, which is involved in lipid metabolism [21, 22]. A recent GWAS about SNPs affecting serum metabolomic traits showed that the *SPTLC3* rs168622 SNP was significantly correlated with hepatic lipid content [19]. Another study conducted in European populations mentioned that a locus (rs3848751) in *SPTLC3* has association with HDL-C and LDL-C in males [23]. However, the association of the *SPTLC3* rs364585 SNP and serum lipid levels and the mechanism were yet unclear. Furthermore, the reproducibility of this association has not been detected in the Chinese population so far.

China is a multi-ethnic country of 56 ethnic groups. Han is the largest ethnic group and Jing is one of the 55 ethnic minorities in south China with a small population of 28199 according to the sixth national census statistics of China in 2010. In the early 16th century, the Jing ancestors emigrated from Vietnam to China, and firstly settled on the three islands of Wanwei, Wutou and Shanxin in Dongxing City, where almost all of the Jing population now live [24]. The Jing population is the only oceanic ethnic group in China and preserves their custom of intra-ethnic marriage, which suggests that there are lots of differences between Jing and Han (as well as the other landlocked nationalities) nationalities in diet custom and culture characteristics. Several previous studies have showed that the associations of variants in several lipid-related genes and serum lipid profiles were significantly different between the Jing and Han populations and their gender subgroups [25–27]. However, to the best of our knowledge, the association between the *SPTLC3* rs364585 SNP and serum lipid levels has not been previously reported in this population. Therefore, the present study was evaluated the association between the rs364585 SNP and several environmental factors with serum lipid levels in the Guangxi Han and Jing populations.

## Methods

### Subjects

A total of 824 unrelated subjects (405 males, 49.15% and 419 females, 50.85%) of Han nationality and 783 unrelated participants (389 males, 49.68% and 394 females, 50.32%) of Jing nationality were randomly selected from our previous stratified randomized samples. All participants were rural agricultural (Han) and/or fishery (Jing) workers living in Jiangping Down, Dongxing City, Guangxi Zhuang Autonomous Region, People’s Republic of China. The participants’ age ranged from 15–80 years with a mean age of 57.27 ± 12.40 years in Han and 57.19 ± 12.50 years in Jing, respectively. All participants were essentially healthy and had no evidence of diseases related to atherosclerosis, CVD and diabetes. Any participant had a history of taking medications known to affect serum lipid levels (lipid-lowering drugs such as statins or fibrates, beta blockers, diuretics, or hormones) was excluded before the blood sample was taken. The study design was approved by the Ethics Committee of the First Affiliated Hospital, Guangxi Medical University. Informed consent was obtained from all participants.

### Epidemiological survey

The survey was carried out using internationally standardized methods, following a common protocol [28]. Information on demographics, socioeconomic status, and lifestyle factors was collected with standardized questionnaires. The intake of alcohol was quantified as the number of liang (about 50 g) of rice wine, corn wine, rum, beer, or liquor consumed during the preceding 12 months. Alcohol consumption was categorized into groups of grams of alcohol per day: 0, < 25 and ≥ 25. Smoking status was categorized into groups of cigarettes per day: 0, < 20 and ≥ 20. In the physical examination, several parameters such as height, weight, and waist circumference were measured. Blood pressure of the subjects in a sitting position was measured taking the mean of 3 separated intevals after the subjects had a 5-min rest using a mercury sphygmomanometer. Body mass index (BMI) was calculated as weight/height^2^ (kg/m^2^).

### Biochemical measurements

A fasting venous blood sample of 5 ml was drawn from the participants. The levels of TC, TG, HDL-C and LDL-C in the samples were determined by enzymatic methods with commercially available kits. Serum ApoA1 and ApoB levels were assessed by the immune-turbidimetric immunoassay [29, 30]. Fasting blood glucose was determined by glucose meter.

### DNA amplification and genotyping

Genomic DNA was isolated from peripheral blood leukocytes using the phenol-chloroform method [31, 32]. The extracted DNA was stored at 4 °C until analysis. The *SPTLC3* rs364585 SNP was genotyped by polymerase chain reaction and restriction fragment length polymorphism (PCR-RFLP). PCR amplification was performed using 5’-TGCCACCTGACCATTTCTCC-3’ as the forward and 5’-AACAAACTTCTGCCTGCCTG-3’ as reversed primer pair. Each amplification reaction was performed in a total volume of 25 μL, 12.5 μL of 2 × *Taq* PCR MasterMix (constituent: 0.1 U *Taq* polymerase/μL, 500 μM dNTP each and PCR buffer), nuclease-free water 8.5 μL, 1 μL each primer (10 pmol/L) and 2 μL genomic DNA, processing started with 5 min of pre-denaturing at 95 °C and followed by 30 s of denaturing at 95 °C, 30 s of annealing at 59 °C and 35 s of elongation at 72 °C for 33 cycles. The amplification was completed by a final extension at 72 °C for 7 min. Following electrophoresis on a 2.0% agarose gel with 0.5 μg/mL ethidium bromide, the amplification products were visualized under ultraviolet light. For the restriction digestion, 5μL of amplified DNA and 5 U of *Mly*I restriction enzyme were added to each reaction mix, and samples were digested at 37 °C for 30 min. After restriction enzyme digestion of the amplified DNA, genotypes were identified by electrophoresis on 2% ethidium bromide stained agarose gels and visualized under ultraviolet light. Six samples (each genotype in two; respectively) detected by the PCR-RFLP were also confirmed by direct sequencing. The PCR products were purified by low melting point gel electrophoresis and phenol extraction, and then the DNA sequences were analyzed using an ABI Prism 3100 (Applied Biosystems) in Shanghai Sangon Biological Engineering Technology & Services Co., Ltd., People’s Republic of China.

### Diagnostic criteria

The normal values of serum TC, TG, HDL-C, LDL-C, ApoA1 and ApoB levels, and the ratio of ApoA1 to ApoB in our Clinical Science Experiment Center were 3.10-5.17, 0.56-1.70, 1.16-1.42, 2.70-3.10 mmol/L, 1.20-1.60, 0.80-1.05g/L, and 1.00-2.50; respectively. The individuals with TC > 5.17 mmol/L and/or TG > 1.70 mmol/L were defined as hyperlipidemic [33, 34]. Hypertension was diagnosed according to the criteria of 1999 World Health Organization-International Society of Hypertension Guide lines for the management of hypertension [35, 36]. The diagnostic criteria of overweight and obesity were according to the Cooperative Meta-analysis Group of China Obesity Task Force. Normal weight, overweight and obesity were defined as a BMI < 24, 24-28, and > 28 kg/m^2^; respectively [37].

### Statistical analysis

The statistical analyses were performed with the statistical software package SPSS 17.0 (SPSS Inc., Chicago, Illinois). The quantitative variables were presented as mean ± standard deviation (serum TG levels were presented as medians and interquartile ranges). Allele frequency was determined via direct counting, and the Hardy-Weinberg equilibrium was verified with the standard goodness-of-fit test. The genotype distribution between the groups was analyzed by the chi-square test. General characteristics between two ethnic groups were compared by the Student’s unpaired *t*-test. The association between genotypes and serum lipid parameters was tested by analysis of covariance (ANCOVA) with age, sex, BMI, cigarette smoking, and alcohol consumption as covariates. Multivariable linear regression analyses with stepwise modeling were used to determine the correlation between genotypes (GG = 1, GA/AA = 2) and several environmental factors with serum lipid levels in males and females of Han and Jing populations. Two sided *P* value < 0.05 was considered statistically significant.

## Results

### General characteristics and serum lipid profiles

The general characteristics and serum lipid profiles between the two ethnic groups and between males and females of the Han and Jing populations are shown in Tables 1 and 2. The values of height, weight, BMI, waist circumference, blood glucose and the levels of TC were higher in Jing than in Han (*P* < 0.05-0.001), whereas the percentage of alcohol consumption, the levels of diastolic blood pressure, LDL-C, and ApoA1 were lower in Jing than in Han (*P* < 0.05-0.001). Subgroup analyses according to sex showed that males had higher height, weight, waist circumference, the percentage of cigarette smoking and alcohol consumption than females in both ethnic groups (*P* < 0.05-0.001). Han males had lower systolic blood pressure, pulse pressure, TC, TG and HDL-C levels than females (*P* < 0.01 for all). Jing men had lower HDL-C and ApoA1 levels and the ApoA1/ApoB ratio than women (*P* < 0.05-0.001). Han males had higher percentage of cigarette smoking and alcohol consumption and serum ApoA1 level and lower values of weight, BMI, waist circumference, pulse pressure, blood glucose and serum TC level than Jing males (*P* < 0.05-0.001). Han females had higher pulse pressure and LDL-C levels and lower values of height, weight, BMI and waist circumference than Jing females (*P* < 0.05-0.001).

**Table 1:** Comparison of demographic, lifestyle characteristics, serum lipid profiles and the genotype and allele frequencies of the *SPTLC3* rs364585 SNP between the Han and Jing populations

Parameter	Han	Jing	*t* (*x* ^2^)	*P*
No. of patients	824	783		
Male/female	405 / 419	389 / 394	0.045	0.832
Age (years)	57.27 ± 12.40	57.19 ± 12.50	0.122	0.903
Height (cm)	157.28 ± 8.27	158.08 ± 7.87	−1.984	0.047
Weight (kg)	56.35 ± 9.41	58.80 ± 10.09	−5.018	0.000
Body mass index (kg/m 2)	22.74 ± 3.19	23.46 ± 3.20	−4.517	0.000
Waist circumference (cm)	77.46 ± 8.90	80.38 ± 9.20	−6.647	0.000
Smoking status (n%)				
0 g/day (non-smoker)	666 (80.8)	656 (83.8)		
≤ 20 cigarettes/day	31 (3.8)	34 (4.3)		
> 20 cigarettes/day	127 (15.4)	93 (11.9)	4.425	0.109
Alcohol consumption (n%)				
0 g/day (non-drinker)	648 (78.6)	681 (87.0)		
≤ 25 g/day	40 (4.9)	83 (10.6)		
> 25 g/day	136 (16.5)	19 (2.4)	103.189	0.000
Systolic blood pressure (mmHg)	132.40 ± 18.90	132.18 ± 21.91	0.215	0.830
Diastolic blood pressure (mmHg)	81.78 ± 10.45	80.34 ± 10.30	2.778	0.006
Pulse pressure (mmHg)	50.62 ± 15.13	51.84 ± 17.81	−1.481	0.139
Glucose (mmol/l)	6.63 ± 1.06	6.86 ± 1.62	−3.390	0.001
Total cholesterol (mmol/l)	4.98 ± 0.85	5.08 ± 0.90	−2.286	0.022
Triglyceride (mmol/l)	1.39(0.67)	1.43(0.75)	−0.349	0.727
HDL-C (mmol/l)	1.80 ± 0.52	1.79 ± 0.46	0.419	0.676
LDL-C (mmol/l)	2.87 ± 0.44	2.82 ± 0.43	2.593	0.010
Apo A1 (g/l)	1.33 ± 0.20	1.28 ± 0.22	4.116	0.000
ApoB (g/l)	1.05 ± 0.24	1.04 ± 0.23	1.023	0.307
ApoA1/ApoB	1.32 ± 0.36	1.29 ± 0.38	1.481	0.139
Genotype [n(%)]				
GG	223(27.1)	235(30.0)		
GA	392(47.6)	371(47.4)		
AA	209(25.4)	177(22.6)	2.501	0.286
Allele [n(%)]				
G	838(50.9)	841(53.7)		
A	810(49.1)	725(46.3)	2.622	0.105

*HDL-C* high-density lipoprotein cholesterol, *LDL-C* low-density lipoprotein cholesterol, *Apo* Apolipoprotein. The value of triglyceride was presented as median (interquartile range), the difference between the two ethnic groups was determined by the Wilcoxon-Mann-Whitney test

**Table 2:** Comparison of demographic, lifestyle characteristics, serum lipid profiles and the genotype and allele frequencies of the *SPTLC3* rs364585 SNP between males and females in the Han and Jing populations

Parameter	Han		*t* (*x* ^2^)	*P*	Jing		*t* (*x* ^2^)	*P*
	Male	Female			Male	Female		
No. of patients	405	419			389	394		
Age (years)	56.88 ± 11.64	57.65 ± 13.10	−0.888	0.375	57.77 ± 14.20	56.64 ± 12.27	1.129	0.234
Height (cm)	162.99 ± 5.56	151.76 ± 6.51	26.538	0.000	162.94 ± 6.60	153.29 ± 5.82^c^	21.728	0.000
Weight (kg)	60.29 ± 8.53	52.54 ± 8.83	12.820	0.000	62.35 ± 10.02^b^	55.30 ± 8.87^c^	10.437	0.000
Body mass index (kg/m^2^)	22.68 ± 2.81	22.80 ± 3.52	−0.560	0.567	23.42 ± 3.10^c^	23.50 ± 3.30^b^	−0.333	0.740
Waist circumference (cm)	78.31 ± 8.19	76.63 ± 9.49	2.730	0.006	81.59 ± 9.83^c^	79.19 ± 8.40^c^	3.677	0.000
Smoking status (n%)								
Non-smoker	248 (61.2)	418 (99.8)			264 (67.9)	392 (99.5)		
Smoker	157 (38.8)	1(0.2)	196.00	0.000	115 (32.1)^a^	2 (0.5)	133.70	0.000
Alcohol consumption (n%)								
Non- drinker	232 (57.3)	416 (99.3)			292(75.1)	389 (98.7)		
Drinker	173 (42.7)	3 (0.7)	216.01	0.000	97(24.9)^c^	5 (1.3)	96.65	0.000
Systolic blood pressure (mmHg)	130.14 ± 16.58	134.58 ± 20.79	−3.392	0.001	132.26 ± 20.77	132.11 ± 23.00	0.096	0.924
Diastolic blood pressure (mmHg)	81.99 ± 10.38	81.58 ± 10.50	0.554	0.580	80.68 ± 10.61	80.02 ± 10.00^a^	0.901	0.368
Pulse pressure (mmHg)	48.16 ± 13.21	53.00 ± 16.44	−4.651	0.000	51.59 ± 16.87^b^	52.09 ± 18.71	−0.403	0.687
Glucose (mmol/l)	6.58 ± 1.08	6.68 ± 1.04	−1.348	0.178	6.94 ± 1.75^c^	6.79 ± 1.49	1.307	0.191
Total cholesterol (mmol/l)	4.90 ± 0.83	5.06 ± 0.87	−2.734	0.006	5.04 ± 0.82^a^	5.11 ± 0.97	−1.165	0.244
Triglyceride (mmol/l)	1.39(1.71)	1.40(1.63)	−2.720	0.007	1.48(1.83)	1.40(1.63)	−0.963	0.336
HDL-C (mmol/l)	1.74 ± 0.55	1.87 ± 0.48	−2.417	0.000	1.75 ± 0.46	1.83 ± 0.45	−2.417	0.016
LDL-C (mmol/l)	2.86 ± 0.43	2.89 ± 0.45	−3.615	0.382	2.82 ± 0.37	2.81 ± 0.48^a^	0.183	0.855
Apo A1 (g/l)	1.33 ± 0.21	1.32 ± 0.20	0.263	0.793	1.26 ± 0.21^c^	1.30 ± 0.23	−2.764	0.006
ApoB (g/l)	1.06 ± 0.23	1.05 ± 0.26	0.465	0.642	1.04 ± 0.22	1.03 ± 0.24	1.011	0.312
ApoA1/ApoB	1.31 ± 0.36	1.33 ± 0.37	−0.697	0.486	1.26 ± 0.40	1.32 ± 0.37	−2.102	0.036
Genotype [n(%)]								
GG	105(25.9)	118(28.2)			108(27.8)	127(32.2)		
GA	188(46.4)	204(48.7)			188(48.3)	183(46.4)		
AA	112(27.7)	97(23.2)	2.250	0.325	93(23.9)	84(21.3)	2.029	0.363
Allele [n(%)]								
G	398(49.1)	440(52.6)			404(51.9)	437(55.5)		
A	412(50.9)	398(47.4)	1.972	0.171	374(48.1)	351(44.5)	1.961	0.161

*HDL-C* high-density lipoprotein cholesterol, *LDL-C* low-density lipoprotein cholesterol, *Apo* Apolipoprotein. The value of triglyceride was presented as median (interquartile range), the difference between the two groups was determined by the Wilcoxon-Mann-Whitney test. ^a^ *P* < 0.001, ^b^ *P* < 0.01 and ^c^ *P* < 0.05 in comparison with the same sex subgroup of the Han ethnic group

### Results of genotyping

**Fig. 1:**
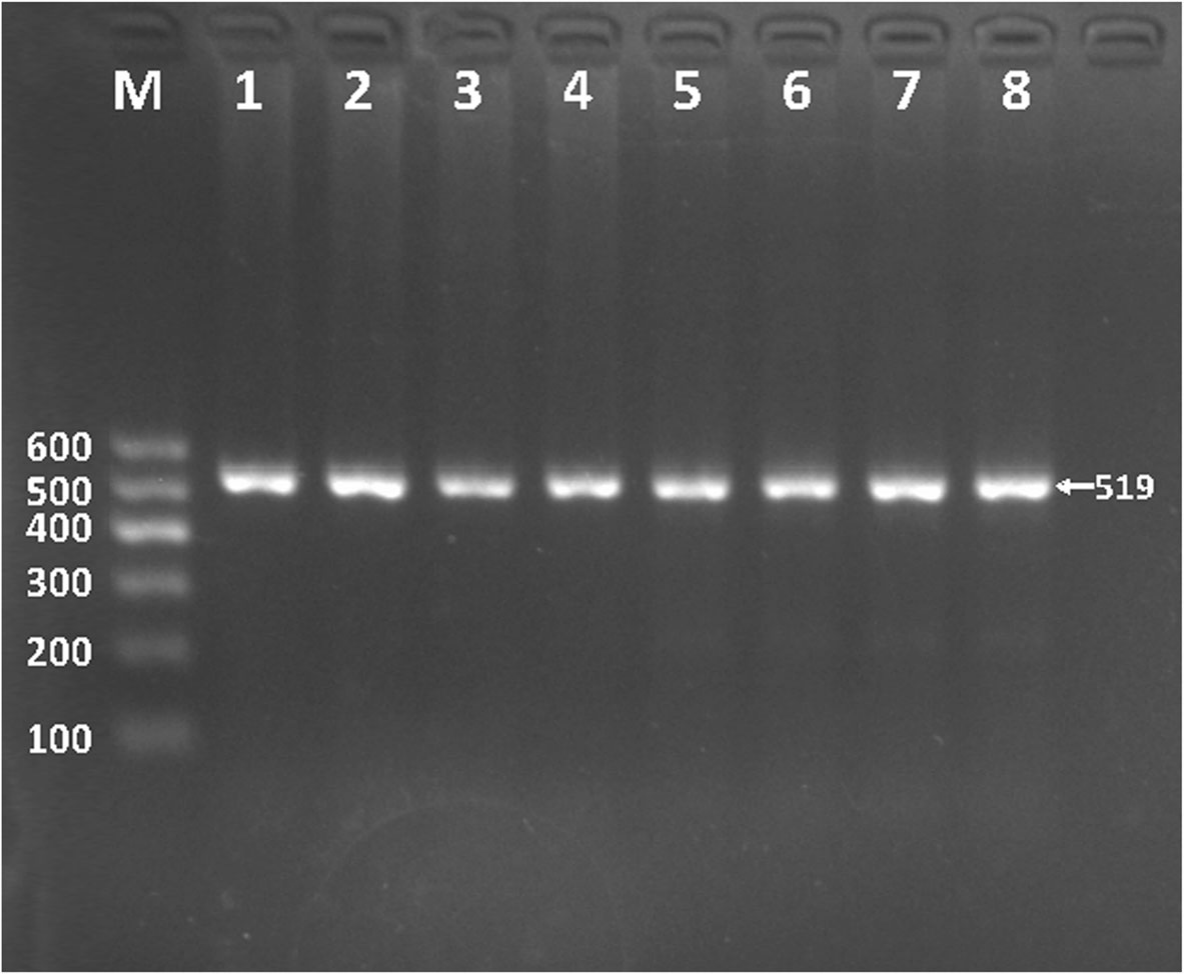
Electrophoresis of polymerase chain reaction products of the samples. Lane M is the 100-bp marker ladder; Lanes 1-8 are samples, the 519-bp bands are the target genes

**Fig. 2:**
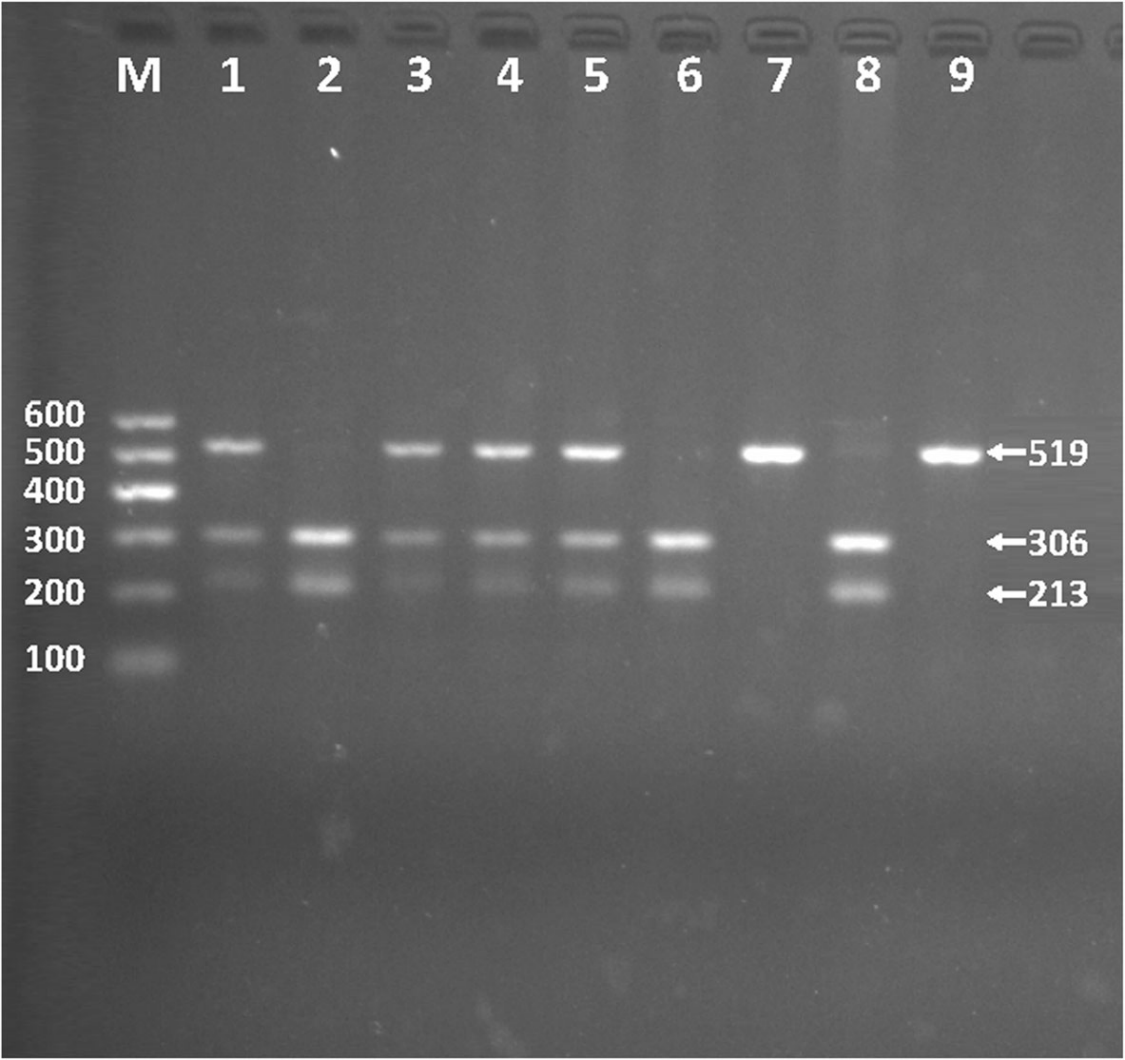
Genotyping of the *SPTLC3* rs364585 SNP. Lane M is the 100-bp Marker Ladder; lanes 2, 6 and 8, GG genotype (306- and 213-bp); lanes 1, 3, 4 and 5, GA genotype (519-, 306- and 213-bp); and lanes 7 and 9, AA genotype (519-bp)

**Fig. 3:**
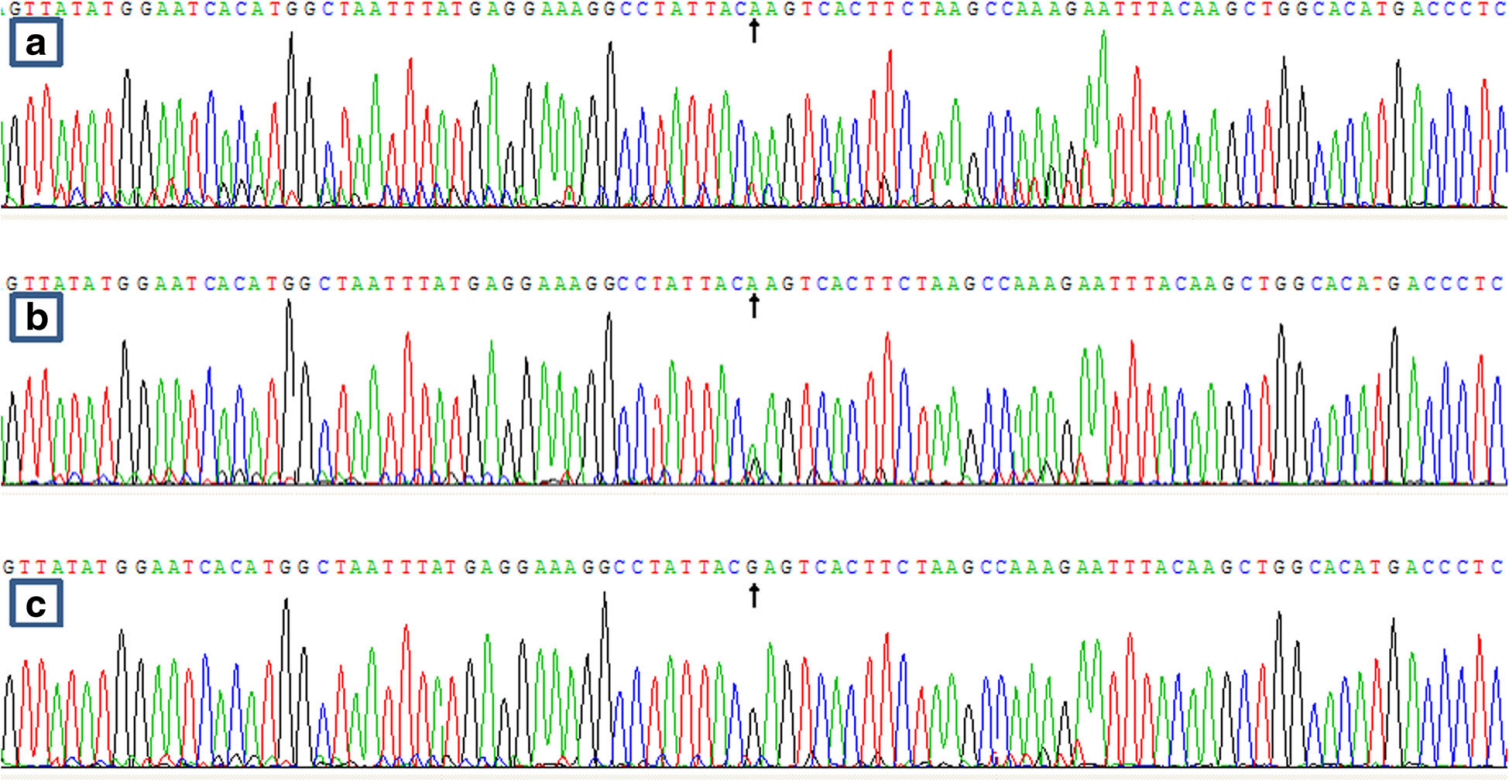
A part of the nucleotide sequence of the *SPTLC3* rs364585 SNP. **a** AA genotype; **b** GA genotype; and **c** GG genotype

After the genomic DNA of the samples was amplified by PCR, the purpose gene of 519-bp nucleotide sequences could be seen in all samples (Fig. 1). The genotypes identified were labeled according to the presence or absence of the enzyme restriction sites. Thus, AA genotype is homozygote for the absence of the site (band at 519-bp), GA genotype is heterozygote for the presence and absence of the site (bands at 519-, 306- and 213-bp) and GG genotype is homozygote for the presence of the site (bands at 306- and 213-bp, Fig. 2). The GG, GA and AA genotypes detected by PCR-RFLP were also confirmed by direct sequencing (Fig. 3).

### Genotypic and allelic frequencies

The genotypic and allelic distribution of the rs364585 SNP is also revealed in Tables 1 and 2. The genotypic distribution was followed Hardy-Weinberg equilibrium (HWE). The frequency of *SPTLC3* rs364585-A allele was 49.1% in Han and 46.3% in Jing. There was no significant difference in either genotypic or allelic frequencies between Han and Jing (Table 1), or between males and females (Table 2) of the both ethnic groups.

### Genotypes and serum lipid levels

Tables 3 and 4 describe the association between genotypes and serum lipid levels in the two ethnic groups. The levels of LDL-C and the ratio of ApoA1 to ApoB in Han were different between the genotypes (*P* < 0.05-0.001), the A allele carriers had higher LDL-C levels and lower the ApoA1/ApoB ratio than the A allele non-carriers. For the Jing population, serum TC, HDL-C and LDL-C levels were different between the genotypes (*P* < 0.05-0.001), the A allele carriers had higher TC and LDL-C levels and lower HDL-C levels than the A allele non-carriers. In the sex subgroup analyses, the A allele carriers in Han males had higher serum LDL-C levels than the A allele non-carriers (*P* < 0.05); the A allele carriers in Jing had higher TC and LDL-C levels in males; and higher LDL-C levels and lower HDL-C levels in females than the A allele non-carriers (*P* < 0.05 for all).

**Table 3:** Comparison of the genotypes and serum lipid levels in the Han and Jing populations

Genotype	n	TC (mmol/L)	TG (mmol/L)	HDL-C (mmol/L)	LDL-C (mmol/L)	ApoA1 (g/L)	ApoB (g/L)	ApoA1 /ApoB
Han								
GG	223	4.96 ± 0.90	1.38(0.69)	1.84 ± 0.46	2.80 ± 0.45	1.33 ± 0.20	1.04 ± 0.25	1.35 ± 0.39
GA/AA	601	4.98 ± 0.83	1.40(0.67)	1.79 ± 0.54	2.89 ± 0.43	1.32 ± 0.20	1.06 ± 0.24	1.31 ± 0.36
*F*		0.796	1.429	2.395	14.391	0.698	1.572	4.243
*P*		0.373	0.153	0.122	0.000	0.404	0.210	0.039
Jing								
GG	235	5.00 ± 0.88	1.40(0.72)	1.85 ± 0.43	2.73 ± 0.46	1.29 ± 0.22	1.04 ± 0.25	1.34 ± 0.46
GA/ AA	548	5.12 ± 0.91	1.44(0.75)	1.77 ± 0.47	2.84 ± 0.43	1.27 ± 0.22	1.04 ± 0.22	1.28 ± 0.35
*F*		6.391	0.062	6.612	16.788	0.734	0.002	2.705
*P*		0.012	0.979	0.013	0.000	0.392	0.961	0.100

*TC* total cholesterol, *TG* triglyceride, *HDL-C* high-density lipoprotein cholesterol, *LDL-C* low-density lipoprotein cholesterol, *ApoA1* apolipoprotein A1, *ApoB* apolipoprotein B, *ApoA1/ApoB* the ratio of apolipoprotein A1 to apolipoprotein B. The value of TG was presented as median (interquartile range), the difference between the genotypes was determined by the Wilcoxon-Mann-Whitney test

**Table 4:** Comparison between the *SPTLC3* rs364585 SNP genotypes and serum lipid levels between males and females in the Han and Jing populations

Ethnic/ Genotype	n	TC (mmol/L)	TG (mmol/L)	HDL-C (mmol/L)	LDL-C (mmol/L)	ApoA1 (g/L)	ApoB (g/L)	ApoA1 /ApoB
Han/male								
GG	105	4.85 ± 0.88	1.32(0.62)	1.80 ± 0.45	2.75 ± 0.41	1.34 ± 0.22	1.03 ± 0.21	1.35 ± 0.38
GA / AA	300	4.92 ± 0.80	1.41(0.72)	1.72 ± 0.57	2.90 ± 0.42	1.32 ± 0.20	1.07 ± 0.23	1.30 ± 0.35
*F*		1.560	1.674	2.028	11.855	1.117	2.088	2.563
*P*		0.212	0.094	0.155	0.001	0.291	0.149	0.110
Han/female								
GG	118	5.07 ± 0.90	1.41(0.68)	1.87 ± 0.46	2.83 ± 0.47	1.32 ± 0.18	1.05 ± 0.29	1.34 ± 0.40
GA / AA	301	5.05 ± 0.86	1.40(0.60)	1.87 ± 0.48	2.93 ± 0.44	1.32 ± 0.20	1.05 ± 0.25	1.33 ± 0.36
*F*		0.004	0.358	0.509	2.434	0.128	0.004	1.096
*P*		0.951	0.720	0.476	0.120	0.721	0.948	0.296
Jing/male								
GG	108	4.86 ± 0.72	1.40(0.95)	1.81 ± 0.42	2.75 ± 0.36	1.29 ± 0.23	1.06 ± 0.23	1.31 ± 0.48
GA/ AA	281	5.12 ± 0.85	1.50(0.81)	1.73 ± 0.48	2.85 ± 0.38	1.25 ± 0.20	1.05 ± 0.21	1.25 ± 0.37
*F*		14.266	0.448	0.278	6.305	1.200	0.616	0.465
*P*		0.000	0.654	0.598	0.012	0.274	0.433	0.496
Jing/female								
GG	127	5.11 ± 0.98	1.40(0.59)	1.92 ± 0.42	2.72 ± 0.53	1.31 ± 0.21	1.02 ± 0.27	1.36 ± 0.44
GA/ AA	267	5.12 ± 0.97	1.39(0.66)	1.79 ± 0.45	2.86 ± 0.48	1.30 ± 0.25	1.04 ± 0.23	1.31 ± 0.33
*F*		0.035	0.669	7.450	6.235	0.018	0.005	1.308
*P*		0.852	0.503	0.007	0.013	0.892	0.945	0.253

*TC* total cholesterol, *TG* triglyceride, *HDL-C* high-density lipoprotein cholesterol, *LDL-C* low-density lipoprotein cholesterol, *ApoA1* apolipoprotein A1, *ApoB* apolipoprotein B, *ApoA1/ApoB* the ratio of apolipoprotein A1 to apolipoprotein B. The values of triglyceride were presented as median (interquartile range), and the difference between the GG and GA/AA genotypes was determined by the Wilcoxon-Mann-Whitney test

### Relative factors for serum lipid parameters

Several environmental factors such as age, gender, height, weight, waist circumference, alcohol consumption and cigarette smoking, and traditional cardiovascular risk factors such as BMI, fasting blood glucose and blood pressure levels were also correlated with serum lipid parameters in the Han and Jing populations and in males and females of both ethnic groups (*P* < 0.05-0.001, Tables 5 and 6).

**Table 5:** The risk factors for serum lipid parameters in the Han and Jing populations

Lipid	Risk factor	B	Std.error	Beta	*t*	*P*
Han and Jing						
TC	Glucose	0.140	0.016	0.220	8.922	0.000
	Age	0.009	0.002	0.130	4.838	0.000
	Gender	0.198	0.047	0.113	4.206	0.000
	Diastolic blood pressure	0.005	0.002	0.056	2.312	0.021
	Genotype	0.097	0.046	0.050	2.091	0.037
	Pulse pressure	−0.003	0.001	−0.060	−2.233	0.026
	Ethnic group	0.102	0.043	0.058	2.369	0.018
	Alcohol consumption	0.077	0.038	0.055	2.034	0.042
TG	Waist circumference	0.038	0.004	0.406	8.415	0.000
	Cigarette smoking	0.257	0.031	0.211	8.403	0.000
	Glucose	0.071	0.015	0.113	4.784	0.000
	Height	−0.015	0.003	−0.143	−4.952	0.000
	Diastolic blood pressure	0.007	0.002	0.085	3.575	0.000
	Body mass index	−0.029	0.012	−0.109	−2.338	0.020
	Age	−0.003	0.002	−0.053	−2.139	0.033
HDL-C	Waist circumference	−0.017	0.001	−0.321	−12.942	0.000
	Alcohol consumption	0.143	0.021	0.183	6.662	0.000
	Gender	0.193	0.034	0.198	5.602	0.000
	Cigarette smoking	−0.053	0.019	−0.076	−2.717	0.007
	Height	0.008	0.002	0.124	3.641	0.000
	Genotype	−0.070	0.025	−0.065	−2.778	0.006
	Age	0.003	0.001	0.070	2.691	0.007
	Ethnic group	0.061	0.024	0.063	2.590	0.010
LDL-C	Glucose	0.037	0.008	0.116	4.650	0.000
	Genotype	0.123	0.024	0.127	5.223	0.000
	Age	0.003	0.001	0.091	3.658	0.000
	Diastolic blood pressure	0.003	0.001	0.076	3.081	0.002
	Ethnic group	−0.056	0.021	−0.064	−2.611	0.009
ApoA1	Waist circumference	−0.003	0.001	−0.137	−3.059	0.002
	Alcohol consumption	0.077	0.009	0.224	8.472	0.000
	Gender	0.052	0.011	0.121	4.553	0.000
	Glucose	−0.011	0.004	−0.068	−2.818	0.005
	Systolic blood pressure	0.001	0.000	0.067	2.721	0.007
	Body mass index	−0.006	0.003	−0.089	−2.006	0.045
ApoB	Waist circumference	0.005	0.001	0.200	7.974	0.000
	Age	0.002	0.000	0.089	3.364	0.001
	Ethnic group	−0.027	0.012	−0.056	−2.299	0.022
	Systolic blood pressure	0.001	0.000	0.061	2.294	0.022
ApoA1/ApoB	Waist circumference	−0.007	0.002	−0.172	−3.447	0.001
	Glucose	−0.023	0.007	−0.084	−3.485	0.001
	Alcohol consumption	0.065	0.016	0.108	4.112	0.000
	Age	−0.002	0.001	−0.059	−2.241	0.025
	Genotype	−0.049	0.020	−0.058	−2.470	0.014
	Weight	−0.039	0.011	−1.020	−3.672	0.000
	Height	0.029	0.008	0.634	3.801	0.000
	Body mass index	0.082	0.026	0.702	3.145	0.002
	Gender	0.063	0.026	0.084	2.414	0.016
Han						
TC	Glucose	0.210	0.027	0.262	7.710	0.000
	Cigarette smoking	−0.117	0.039	−0.101	−3.042	0.002
	Pulse pressure	0.001	0.000	0.072	2.141	0.033
	Systolic blood pressure	0.003	0.002	0.073	2.123	0.034
TG	Waist circumference	0.038	0.006	0.386	6.678	0.000
	Cigarette smoking	0.226	0.041	0.190	5.535	0.000
	Glucose	0.107	0.027	0.131	3.978	0.000
	Diastolic blood pressure	0.007	0.003	0.089	2.639	0.008
	Weight	−0.014	0.005	−0.150	−2.558	0.011
HDL-C	Waist circumference	−0.015	0.002	−0.265	−7.742	0.000
	Cigarette smoking	−0.125	0.028	−0.177	−4.404	0.000
	Alcohol consumption	0.135	0.028	0.196	4.803	0.000
	Gender	0.212	0.051	0.205	4.162	0.000
	Height	0.009	0.003	0.138	3.008	0.003
	Genotype	−0.089	0.038	−0.076	−2.306	0.021
LDL-C	Glucose	0.073	0.014	0.175	5.042	0.000
	Systolic blood pressure	0.003	0.001	0.133	3.853	0.000
	Genotype	0.122	0.033	0.123	3.658	0.000
ApoA1	Alcohol consumption	0.071	0.009	0.264	7.538	0.000
	Weight	−0.004	0.001	−0.186	−5.318	0.000
ApoB	Waist circumference	0.005	0.001	0.185	5.415	0.000
	Systolic blood pressure	0.002	0.000	0.132	3.763	0.000
	Glucose	0.019	0.008	0.083	2.393	0.017
ApoA1/ApoB	Body mass index	−0.028	0.004	−0.245	−7.259	0.000
	Glucose	−0.042	0.012	−0.122	−3.570	0.000
	Alcohol consumption	0.063	0.019	0.130	3.376	0.001
	Gender	0.078	0.028	0.107	2.775	0.006
	Systolic blood pressure	−0.002	0.001	−0.080	−2.290	0.022
	Genotype	−0.056	0.027	−0.069	−2.065	0.039
Jing						
TC	Glucose	0.103	0.019	0.186	5.414	0.000
	Age	0.017	0.003	0.262	6.767	0.000
	Pulse pressure	−0.008	0.002	−0.151	−4.064	0.000
	Gender	0.348	0.073	0.193	4.754	0.000
	Cigarette smoking	0.198	0.053	0.146	3.767	0.000
	Alcohol consumption	0.296	0.077	0.139	3.825	0.000
	Body mass index	0.056	0.017	0.198	3.257	0.001
	Genotype	0.166	0.066	0.085	2.524	0.012
	Waist circumference	−0.015	0.006	−0.158	−2.539	0.011
	Diastolic blood pressure	0.006	0.003	0.069	1.964	0.050
TG	Waist circumference	0.034	0.003	0.373	10.642	0.000
	Cigarette smoking	0.286	0.045	0.226	6.436	0.000
	Height	−0.016	0.004	−0.149	−4.041	0.000
	Glucose	0.049	0.017	0.096	2.896	0.004
HDL-C	Waist circumference	−0.018	0.002	−0.364	−11.101	0.000
	Alcohol consumption	0.250	0.037	0.232	6.739	0.000
	Gender	0.103	0.032	0.113	3.259	0.001
	Genotype	−0.089	0.032	−0.090	−2.758	0.006
LDL-C	Genotype	0.136	0.033	0.144	4.123	0.000
	Age	0.005	0.001	0.149	4.028	0.000
	Cigarette smoking	0.090	0.026	0.139	3.503	0.000
	Gender	0.080	0.034	0.092	2.308	0.021
	Glucose	0.019	0.009	0.073	2.054	0.040
	Diastolic blood pressure	0.003	0.001	0.071	2.031	0.043
ApoA1	Waist circumference	−0.006	0.001	−0.261	−7.425	0.000
	Alcohol consumption	0.067	0.019	0.127	3.469	0.001
	Gender	0.048	0.016	0.107	2.933	0.003
	Systolic blood pressure	0.001	0.000	0.079	2.278	0.023
ApoB	Waist circumference	0.005	0.001	0.202	5.802	0.000
	Age	0.002	0.001	0.117	3.348	0.001
ApoA1/ApoB	Waist circumference	−0.008	0.003	−0.196	−3.198	0.001
	Age	−0.003	0.001	−0.122	−3.482	0.001
	Alcohol consumption	0.087	0.031	0.095	2.776	0.006
	Weight	−0.005	0.002	−0.136	−2.187	0.029

*TC* total cholesterol, *TG* triglyceride, *HDL-C* high-density lipoprotein cholesterol, *LDL-C* low-density lipoprotein cholesterol, *ApoA1* apolipoprotein A1, *ApoB* apolipoprotein B, *ApoA1/ApoB* the ratio of apolipoprotein A1 to apolipoprotein B

**Table 6:** The risk factors for serum lipid parameters in the males and females of the Han and Jing populations

Lipid	Risk factor	B	Std.error	Beta	*t*	*P*
Han/male						
TC	Glucose	0.224	0.036	0.295	6.291	0.000
	Pulse pressure	0.001	0.000	0.114	2.444	0.015
	Diastolic blood pressure	0.009	0.004	0.110	2.357	0.019
	Cigarette smoking	−0.089	0.042	−0.100	−2.123	0.034
TG	Waist circumference	0.049	0.010	0.405	4.765	0.000
	Cigarette smoking	0.219	0.052	0.201	4.182	0.000
	Glucose	0.168	0.044	0.181	3.770	0.000
	Diastolic blood pressure	0.014	0.005	0.142	2.998	0.003
	Age	−0.013	0.004	−0.146	−2.823	0.005
	Weight	−0.023	0.010	−0.198	−2.256	0.025
HDL-C	Waist circumference	−0.021	0.003	−0.307	−6.068	0.000
	Alcohol consumption	0.129	0.031	0.217	4.171	0.000
	Cigarette smoking	−0.128	0.031	−0.213	−4.164	0.000
	Genotype	−0.138	0.059	−0.111	−2.347	0.019
	Height	0.011	0.005	0.109	2.244	0.025
	Diastolic blood pressure	0.005	0.003	0.101	2.061	0.040
LDL-C	Glucose	0.095	0.019	0.241	5.060	0.000
	Genotype	0.165	0.046	0.170	3.570	0.000
	Diastolic blood pressure	0.005	0.002	0.111	2.324	0.021
ApoA1	Alcohol consumption	0.082	0.011	0.366	7.783	0.000
	Waist circumference	−0.005	0.001	−0.196	−4.153	0.000
ApoB	Waist circumference	0.005	0.001	0.184	3.767	0.000
	Systolic blood pressure	0.002	0.001	0.179	3.513	0.000
	Glucose	0.031	0.011	0.146	2.913	0.004
	Age	−0.002	0.001	−0.118	−2.296	0.022
ApoA1/ApoB	Waist circumference	−0.011	0.002	−0.246	−5.086	0.000
	Alcohol consumption	0.087	0.019	0.221	4.512	0.000
	Systolic blood pressure	−0.003	0.001	−0.153	−2.986	0.003
	Age	0.005	0.002	0.166	3.246	0.001
	Glucose	−0.045	0.017	−0.133	−2.692	0.007
Han/female						
TC	Glucose	0.223	0.039	0.266	5.645	0.000
TG	Waist circumference	0.024	0.004	0.320	6.964	0.000
	Glucose	0.094	0.032	0.135	2.936	0.004
HDL-C	Body mass index	−0.034	0.006	−0.253	−5.330	0.000
LDL-C	Glucose	0.057	0.021	0.131	2.684	0.008
	Age	0.005	0.002	0.156	3.209	0.001
ApoA1	Body mass index	−0.009	0.003	−0.153	−3.170	0.002
ApoB	Height	0.006	0.001	0.215	4.562	0.000
	Age	0.003	0.001	0.166	3.349	0.001
	Height	−0.005	0.002	−0.129	−2.580	0.010
ApoA1/ApoB	Age	−0.003	0.001	−0.113	−2.330	0.020
	Glucose	−0.052	0.016	−0.147	−3.208	0.001
	Body mass index	0.193	0.049	1.846	3.928	0.000
	Height	0.074	0.015	1.311	4.827	0.000
	Weight	−0.095	0.021	−2.293	−4.464	0.000
Jing/male						
TC	Alcohol consumption	0.313	0.072	0.212	4.356	0.000
	Glucose	0.073	0.023	0.156	3.185	0.002
	Cigarette smoking	0.154	0.050	0.159	3.097	0.002
	Age	0.017	0.003	0.292	5.273	0.000
	Genotype	0.315	0.087	0.172	3.634	0.000
	Pulse pressure	−0.011	0.003	−0.232	−4.453	0.000
	Body mass index	0.097	0.026	0.368	3.800	0.000
	Waist circumference	−0.024	0.008	−0.284	−2.857	0.005
TG	Waist circumference	0.040	0.005	0.426	8.633	0.000
	Cigarette smoking	0.214	0.052	0.198	4.119	0.000
	Height	−0.025	0.007	−0.179	−3.484	0.001
	Age	−0.011	0.003	−0.168	−3.249	0.001
	Glucose	0.066	0.025	0.125	2.666	0.008
HDL-C	Waist circumference	−0.019	0.002	−0.402	−8.995	0.000
	Alcohol consumption	0.259	0.037	0.310	6.939	0.000
LDL-C	Cigarette smoking	0.061	0.022	0.139	2.778	0.006
	Genotype	0.091	0.042	0.109	2.161	0.031
	Body mass index	0.012	0.006	0.101	2.001	0.046
ApoA1	Waist circumference	−0.008	0.001	−0.357	−7.594	0.000
	Alcohol consumption	0.072	0.018	0.191	4.056	0.000
ApoB	Weight	0.007	0.001	0.314	6.223	0.000
	Age	0.002	0.001	0.112	2.228	0.026
ApoA1/ ApoB	Waist circumference	−0.015	0.002	−0.375	−7.981	0.000
	Alcohol consumption	0.109	0.034	0.150	3.184	0.002
Jing/female						
TC	Glucose	0.163	0.031	0.251	5.250	0.000
	Age	0.021	0.004	0.266	5.145	0.000
	Pulse pressure	−0.006	0.003	−0.106	−2.046	0.041
TG	Waist circumference	0.026	0.004	0.291	6.007	0.000
	Cigarette smoking	1.164	0.318	0.174	3.661	0.000
	Height	−0.027	0.006	−0.207	−4.240	0.000
HDL-C	Waist circumference	−0.018	0.003	−0.347	−7.170	0.000
	Genotype	−0.131	0.045	−0.138	−2.920	0.004
	Diastolic blood pressure	0.004	0.002	0.099	2.041	0.042
LDL-C	Genotype	0.123	0.050	0.119	2.463	0.014
	Age	0.008	0.002	0.210	4.293	0.000
	Glucose	0.045	0.016	0.139	2.855	0.005
ApoA1	Body mass index	−0.011	0.004	−0.159	−3.194	0.002
ApoB	Age	0.004	0.001	0.191	3.893	0.000
	Body mass index	0.010	0.004	0.138	2.805	0.005
ApoA1/ApoB	Body mass index	−0.023	0.005	−0.205	−4.176	0.000
	Age	−0.004	0.001	−0.144	−2.943	0.003

*TC* total cholesterol, *TG* triglyceride, *HDL-C* high-density lipoprotein cholesterol, *LDL-C* low-density lipoprotein cholesterol, *ApoA1* apolipoprotein A1, *ApoB* apolipoprotein B, *ApoA1/ApoB* the ratio of apolipoprotein A1 to apolipoprotein B

## Discussion

In the present study, we found that the levels of TC were higher but the levels of LDL-C and ApoA1 were lower in Jing than in Han. In the sex subgroup analyses, Han males had lower serum TC, TG and HDL-C levels than Han females, whereas Jing men had lower HDL-C and ApoA1 levels and the ApoA1/ApoB ratio than Jing women. Han males had higher ApoA1 levels and lower TC levels than Jing males, whereas Han females had higher LDL-C levels than Jing females. It was widely realized that dyslipidemia as a serious risk factor for CVD is a multifactorial and complicated disease caused by genetic factors, including lipid-associated gene variants and environmental factors, including age, sex, diet, alcohol consumption, cigarette smoking, obesity, exercise, hypertension [38, 39], and their interactions [12, 13].

Han nationality is the largest ethnic group among the 56 ethnic groups in China and is widely distributed in 2/3 regions of China. Its economy was dominated by agriculture and it abounds in rice, corn and wheat. Jing, one of the 55 official ethnic minorities in China is the only Chinese minority for coastal fisheries and is the only sea people in China. They live in a relatively isolated environment and share local similar recipes. In this case, it has a very special lifestyle and dietary habits compared with the other landlocked nationalities. Their marriages were family-arranged in the old days when they sing antiphonal songs to look for the other half. After antiphonal singing, if the boy’s into the girl he would kick sand toward her while approaching her. If the girl feel the same she would kick back, which means engagement. While the formal engagement ceremony and wedding they need pork, cake, tea, wine, glutinous rice as gifts. Jing stays endogamy, intermarriage with Han or Zhuang people is seldom happened. Owing to its own strict intra-ethnic marriage customs and unique traditions, we speculate that some hereditary characteristics and genotypes of lipid metabolism-related genes in this population might be different from those in Han Chinese.

The genotypic and allelic frequencies of the *SPTLC3* rs364585 SNP in diverse racial/ethnic groups are significantly different. According to the International HapMap Project’s data-base, the frequencies of A allele and AA, AG genotypes were 34.4%, 15.6% and 37.8% in Han Chinese in Beijing; 40.8%, 13.3% and 55.0% in European; 48.9%, 27.3% and 43.2% in Japanese; and 10.8%, 1.7% and 18.3% in Yoruba; respectively. In the present study, we showed that the frequencies of A allele and AA, AG genotypes were 49.1%, 25.4% and 47.6% in Han; and 46.3%, 22.6% and 47.4% in Jing; respectively. There were no conspicuous differences in the genotypic and allelic frequencies of the rs364585 SNP between the Jing and Han populations, or between males and females in the both ethnic groups. As compared with the data in the International HapMap Project’s data-base, we found that the frequencies of the A allele and AA, AG genotypes in our study populations were higher than those in Han Chinese from Beijing, which may be caused by different sample sizes and regions (Beijing vs. Guangxi).

There were hardly any previous studies presented the direct relationship between the *SPTLC3* rs364585 SNP and blood lipid levels in humans except a newly GWAS which showed that the *SPTLC3* rs364585 SNP was significant associated with LDL-C concentrations (*P* < 5 × 10^−8^) in the population of European descent [40]. In the present study, we found that the *SPTLC3* rs364585 SNP was significant associated with multiple serum lipid parameters in our study populations. The A allele carriers had higher LDL-C levels and lower the ApoA1/ApoB ratio in Han; and higher TC and LDL-C levels and lower HDL-C levels in Jing than the A allele non-carriers. When serum lipid parameters were analyzed according to gender, we found that the A allele carriers had higher LDL-C levels in Han males; higher TC and LDL-C levels in Jing males; and higher LDL-C levels and lower HDL-C levels in Jing females than the A allele non-Carriers. These results indicated that the association of the *SPTLC3* rs364585 SNP and serum lipid levels may have racial/ethnic and/or sex specificity.

SPTLC3 is a 552 amino acid single-pass membrane protein and is a member of the class-II pyridoxal-phosphate-dependent amino transferase family. *SPTLC3* encodes a functional subunit of the SPT enzyme-complex that catalyzes the first and rate-limiting step of de novo sphingolipid synthesis [21], which is involved in lipid metabolism. Direct experimental evidence indicates a role for sphingolipids in several common complex chronic disease processes including atherosclerotic plaque formation, myocardial infarction, cardiomyopathy, pancreatic beta cell failure, insulin resistance and type 2 diabetes mellitus [41]. A genome-wide analysis identified that the *SPTLC3* played a functional role in the hepatic lipid accumulation, which is a common example of imbalance in lipid and energy homeostasis [19]. Another study demonstrated that the SNP of rs3848751 in *SPTLC3* was associated with HDL-C and LDL-C in males and variants (rs3848751 and rs6078888) within *SPTLC3* had influence on the risk of myocardial infarction [23]. The results may be due to mutations causing changes in circulating sphingolipid concentrations. Taken together, we speculate that the *SPTLC3* rs364585 mutation may act in the subunit of the SPT enzyme-complex to influence the de novo sphingolipid synthesis and bring about the cascade of events in lipid metabolism. However, the biological function and detailed role of the *SPTLC3* rs364585 SNP in lipid metabolism need to be further explored.

The gonadal hormone is also a contribution factor on lipid metabolism although the reasons for sex differences in serum lipid levels are still unclear. It is commonly accepted that androgens induce changes in serum lipid concentrations that would predispose towards CVD, while oestrogens have the opposite effects [42]. Oestrogens share structural similarities with vitamin E and other lipophilic antioxidants and are thus able to function as scavengers for lipid peroxyl radicals and interrupt the chain reaction of lipid peroxidation. Oestrogens also protect HDL from oxidation, an effect that should preserve the beneficial functions of HDL. In the present study, we found the levels of HDL-C in Han was lower in males than in females and the levels of HDL-C, ApoA1 and the ApoA1/ApoB ratio in Jing were lower in males than females. It is well known that both HDL-C and ApoA1 are protective factors for CVD and the ratio of ApoA1 to ApoB reflects the cholesterol balance between antiatherogenic and atherogenic lipoprotein particles. These results are consistent with previous findings that females have more favorate serum lipid profiles to reduce the risk of CVD than males. In addition, we also found sex difference in the association of the *SPTLC3* rs364585 SNP and serum lipid levels. The reasons for the discrepancy are still not well understood. Other unknown genetic factors may also be involved in determining this complex status. Besides, the sample size may be not large enough to detect the association of the *SPTLC3* rs364585 SNP and serum lipid levels in the subgroup analyses. Further studies are needed to clarify.

Furthermore, it is well recognized that environmental factors such as dietary patterns, lifestyle and physical inactivity are all strongly related with serum lipid levels [39, 43]. In the present study, multiple linear regression analysis showed that serum lipid parameters were also affected by several environmental factors such as age, gender, BMI, waist circumference, alcohol consumption, cigarette smoking, blood pressure and blood glucose. These data suggest that the environmental factors also play an important role in determining serum lipid profiles in our study populations. Fishery is the major source of income for Jing population and fish is appeared most frequently dish on their tables. A kind of fish sauce called nuoc-mam is also popular on Jing people’s dinner table, which contains 17 amino acids (8 essential amino acids included of course). Fish rich in omrga-3 polyunsaturated fatty acids (N-3PUFA) have been suggested to have a favorable effect on serum concentrations of pleiotropic lipid traits. However, previous researches also demonstrated the effects of N-3PUFA on key metabolic functions, including significant rise in TC, TG and LDL-C levels and decrease in HDL-C levels [44, 45].

In addition, we also found that the levels of weight and BMI were higher in Jing than in Han and the percentages of alcohol consumption were lower in Jing males than in Han males (*P* < 0.05 for each). A previous study reported that for every 1-kg decrease in body weight, TG decreased by 0.011 mmol/L and HDL-C increased by 0.011 mmol/L [46]. In another study, Rimm et al. documented that consuming of 30 g of ethanol per day increased the concentrations of HDL-C by 3.99 mg/ dL, ApoA1 by 8.82 mg/dL, and TG by 5.69 mg/ dL [47]. What’s more, Yin et al. also showed that BMI and alcohol consumption could interact with certain lipid-related gene variants to modify the serum lipid levels in Bai Ku Yao and Han Chinese ethnic groups. Therefore, the results of exposure to different environmental factors may further modify the effect of genetic variation on serum lipid levels in our study populations.

There are several major strengths in our study. First, the study is an investigation of a representative random sample of the Jing population, which retains its regional and special customs in China and may be a useful subgroup for population genetic studies. Second, the sample size of the study is moderate with 783 subjects of Jing and 824 subjects of Han Chinese. Third, a recent GWAS has reported the association between the *SPTLC3* rs364585 SNP and serum LDL-C levels [40] and our present study is the first replication of GWAS signals in the Chinese population to provide significant evidence for the association of the *SPTLC3* rs364585 SNP with serum lipid traits. To interpret the findings, however, several potential limitations in our study should be acknowledged. Firstly, we were not able to alleviate the effect of diet and several environmental factors during the statistical analysis. Secondly, although we have detected the effects of the *SPTLC3* rs364585 SNP on serum lipid levels in this study, there are still many lipid-related SNPs and the interactions of SNP-SNP and/or SNP-environmental factors. Finally, we recognize the limited power to provide a more significant advance in understanding the full impact of rs364585 SNP on lipoprotein metabolism. To confirm our findings, further in-depth studies on the biological actions of *SPTLC3* rs364585 variation and the interactions of gene-environment are necessary.

## Conclusions

This study showed that the association of the *SPTLC3* rs364585 SNP and serum lipid profiles is different between the Jing and Han populations and between males and females in the both ethnic groups. These results suggest that there may be a racial/ethnic- and/ or sex-specific association of the *SPTLC3* rs364585 SNP and serum lipid parameters.

## Abbreviations

ANCOVA: Analysis of covariance
Apo: Apolipoprotein
BMI: Body mass index
CVD: Cardiovascular disease
GWAS: Genome-wide association study
HDL-C: High-density lipoprotein cholesterol
HWE: Hardy-Weinberg equilibrium
LDL-C: Low-density lipoprotein cholesterol
N-3PUFA: Omrga-3 polyunsaturated fatty acids
PCR: Polymerase chain reaction
RFLP: Restriction fragment length polymorphism
SNP: Single nucleotide polymorphism
SPTLC3: The serine palmitoyl-transferase long chain base subunit 3
TC: Total cholesterol
TG: Triglyceride
